# Correspondence

**Published:** 1908-03

**Authors:** Edw. C. Cartledge


### CORRESPONDENCE

---

*Editor Journal-Record of Medicine.*

In your January issue you write editorial "Rose Water," referring to a trial before Recorder Judge Broyles against some druggist for selling Jamaica or Tincture of Ginger, upon which some person had been made drunk.

From the newspaper reports it seemed that the druggist put the Recorder and City Attorney to confusion by saying that Tr. Gelsemii and Rose Water and quite all liquid medicines had more or less alcohol to produce intoxication, and belong in a class along with Tr. Ginger and therefore outlawed by our recent prohibition law. Not. so. The law reads: "those things which, if drunk to excess, will produce intoxication." Now, the plain truth is that long before intoxication could be produced by the Trs. and Fl. Exts., heavy in alcohol, other dangers and upsettings would come quick. The only official drugs in common use that can be attacked by the Prohibition law are Tr. Ginger and Elixir Aromatic as occurs to me. Therefore, I think it due the truth to correct the charge that: "The haste and unscientific spirit of the prohibitionists" have outlawed Rose Water, Tr. Gelsemii, etc., as was advertised by daily press and referred to by you.

Yours fraternally,

EDW. C. CARTLEDGE.

